# Literary Notes

**Published:** 1909-08-28

**Authors:** 


### LITERARY NOTES.

The Francis Galton Laboratory of the University of London has published the second issue (Part III.) of "The Treasury of Human Inheritance." Among the fresh features are articles on Insanity by Dr. Urquhart, and on Hermaphroditism by Dr. Bullock.

A correspondent has taken us to task for our recent assertion that English medical journalism is better of its kind than English medical literature. He writes: "I enclose three journals for your inspection. All relate to the same special subject, but one belongs to this country, the second to France, while the third is German. I think any judge of the question would agree that the only point in which our representative surpasses the others is in the number and variety of the advertisement pages. On the other hand, both foreign journals give long lists of recent publications in several languages bearing on the subject in question, while the English one does not. The strongly popular tone of the latter, too, and the curtailing of its scientific articles, make it appear beside the others something like a parish magazine." We would recommend to our correspondent comparison of the leading medical weeklies in this country and on the Continent, whence it will appear that the British medical editor's labours in the way of collecting and presenting information on current questions stand easily first.

Messrs. J. and A. Churchill are about to publish the following new books on medicine and science: "A Text Book of Nervous Diseases." By W. Aldren Turner, M.D., Physician and Lecturer on Neurology, King's College Hospital, and T. Grainger Stewart, M.B., Assistant Physician National Hospital for Paralysis and Epilepsy. This work will cover the whole range of nervous disorders and their treatment, and will be profusely and originally illustrated.—"A Text Book of Experimental Physiology." By N. H. Alcock, M.D., and F. O'Brien Ellison, M.D., St. Mary's Hospital Medical School. With an introduction by E. H. Starling, F.R.S., Jodrell Professor of Physiology University of London. This book, which will be a small one, is designed to meet the need felt for a small handbook on the subject. It will contain thirty-six illustrations.—"A Course of Practical Chemistry," for Use in Public Schools. By A. Beresford Ryley, Science Master Malvern College.—"Sight Testing Made Easy." By W. W. Hardwicke, M.D. This small work will be illustrated with diagrams elucidating the text.—"A Guide to the Paris Medical School." Second Edition. By A. A. Warden, M.D., Physician to Hertford British Hospital, Paris.

